# Exploring the effects of deep brain stimulation and vision on tremor in Parkinson’s disease - benefits from objective methods

**DOI:** 10.1186/s12984-020-00677-3

**Published:** 2020-04-25

**Authors:** Per-Anders Fransson, Maria H. Nilsson, Diederick C. Niehorster, Marcus Nyström, Stig Rehncrona, Fredrik Tjernström, Måns Magnusson, Rolf Johansson, Mitesh Patel

**Affiliations:** 1 Department of Clinical Sciences, Lund University, S-221 85 Lund, Sweden; 2 Department of Health Sciences, Lund University, S-221 85 Lund, Sweden; 3 Memory Clinic, Skåne University Hospital, S-212 24 Malmö, Sweden; 4 Clinical Memory Research Unit, Faculty of Medicine, Lund University, S-221 85 Lund, Sweden; 5 Lund University Humanities Lab, Lund University, S-221 00 Lund, Sweden; 6 Department of Psychology, Lund University, S-221 00 Lund, Sweden; 7 Lund University Cognitive Science, Lund University, S-221 00 Lund, Sweden; 8 Department of Neurosurgery, Lund University, S-221 85 Lund, Sweden; 9 Department of Automatic Control, Lund University, S-221 00 Lund, Sweden; 10 Division of Brain Sciences, Imperial College London, London, W6 8RF UK; 11 Faculty of Science and Engineering, University of Wolverhampton, Wolverhampton, WV1 1LZ UK

**Keywords:** Parkinson’s disease, Tremor, Deep brain stimulation, Subthalamic nucleus

## Abstract

**Background:**

Tremor is a cardinal symptom of Parkinson’s disease (PD) that may cause severe disability. As such, objective methods to determine the exact characteristics of the tremor may improve the evaluation of therapy. This methodology study aims to validate the utility of two objective technical methods of recording Parkinsonian tremor and evaluate their ability to determine the effects of Deep Brain Stimulation (DBS) of the subthalamic nucleus and of vision.

**Methods:**

We studied 10 patients with idiopathic PD, who were responsive to _L_-Dopa and had more than 1 year use of bilateral subthalamic nucleus stimulation. The patients did not have to display visible tremor to be included in the study. Tremor was recorded with two objective methods, a force platform and a 3 dimensional (3D) motion capture system that tracked movements in four key proximal sections of the body (knee, hip, shoulder and head). They were assessed after an overnight withdrawal of anti-PD medications with DBS ON and OFF and with eyes open and closed during unperturbed and perturbed stance with randomized calf vibration, using a randomized test order design.

**Results:**

Tremor was detected with the Unified Parkinson’s Disease Rating Scale (UPDRS) in 6 of 10 patients but only distally (hands and feet) with DBS OFF. With the force platform and the 3D motion capture system, tremor was detected in 6 of 10 and 7 of 10 patients respectively, mostly in DBS OFF but also with DBS ON in some patients. The 3D motion capture system revealed that more than one body section was usually affected by tremor and that the tremor amplitude was non-uniform, but the frequency almost identical, across sites. DBS reduced tremor amplitude non-uniformly across the body. Visual input mostly reduced tremor amplitude with DBS ON.

**Conclusions:**

Technical recording methods offer objective and sensitive detection of tremor that provide detailed characteristics such as peak amplitude, frequency and distribution pattern, and thus, provide information that can guide the optimization of treatments. Both methods detected the effects of DBS and visual input but the 3D motion system was more versatile in that it could detail the presence and properties of tremor at individual body sections.

## Introduction

Tremor in Parkinson’s disease (PD) may cause severe disability, and it can be problematic clinically because sometimes it responds poorly to dopamine replacement therapy [1]. In PD, an insufficient formation and action of dopamine in the substantia nigra pars compacta causes defective transmission of impulses from the basal ganglia to other neuronal structures, thereby producing motor symptoms [2, 3]. The neuronal activity from basal ganglia structures and the thalamus shows rhythmic activity related to tremor [4], suggesting that Parkinsonian tremor arises from central oscillators [5]. To gain further insights into tremor generation and presentation, non-invasive techniques - such as motion capture – have been employed [6–8]. The development of advanced recording systems and signal processing algorithms over the last few decades has provided methods that are more sensitive at detecting tremor. Some key advantages of technical recording methods are their ability to objectively and repeatedly provide precise and detailed information about the characteristics of tremor (e.g., peak amplitude, frequency) compared to subjective visual inspection. For example, the higher accuracy and precision of modern motion capture systems may be utilized in research and in clinical settings to evaluate and customize treatment of tremor [6–8].

Effective treatments for refractory tremor are either surgical lesioning or deep brain stimulation (DBS) in the thalamus, subthalamic nucleus (STN) or globus pallidus internus (GPI) [9]. STN-DBS significantly reduces tremor amplitude [10] and the need for anti-PD medication, which further reduces the motor complications from dopamine therapy [11]. Consequently, here we explore the characteristics of tremor in a small number of well-defined PD patients who were treated with DBS-STN to mitigate PD symptoms.

Furthermore, we wanted to compare the outcomes from a 3D motion capture system against a force platform system. Force platforms are more common in clinical settings than 3D motion systems, but few employ appropriate posturography tests and perform sophisticated analytics of force platform data to explore its full potential. A force platform records the total projected forces and torques produced by the entire body, and thus, the body’s movement of inertia may act as a mechanical filter of tremor within the typical PD frequency range. Moreover, tremor situated in distal parts of the body may not be recordable by a force platform. A 3D motion capture system together with appropriate analytical packages, however, have the capacity to detail the characteristics of tremor simultaneously in both distal and proximal body sections.

The aim of this study was to investigate the utility of a 3D motion capture system and a posturography force platform for detecting tremor in patients with PD. Specifically, we wanted to determine if both methods could quantify and provide novel information about the effects of DBS-STN (without anti-PD medication) and visual input (eyes open / eyes closed) on the amplitude and peak frequency of tremor.

## Materials and methods

### Patients

The investigated population included 10 patients with idiopathic PD, who were responsive to _L_-Dopa and had more than 1 year of use of bilateral subthalamic nucleus (STN) stimulation. The patients did not have to display visible tremor to be included in the study as we aimed to explore the sensitivity of two different technical methods and compare their results to the conventional manual Unified Parkinson’s disease Rating Scale (UPDRS)). Descriptive information (e.g., L-dopa equivalents and DBS parameter settings) is provided in Table 1. The neurosurgical procedure for this population has been described previously elsewhere [14, 15]. All patients were recruited from the Department of Neurosurgery, Skåne University Hospital.

**Table 1:** Patient characteristics

Patients’ characteristics		Median (range)
Gender	9 men, 1 woman	
Age (years)		65 (59–69)
Duration of disease (years)		18 (10–22)
L-dopa equivalent dose (mg/day)		416 (294–989)
Duration of DBS treatment (months)		37 (15–70)
DBS parameter settings	Right: - Amplitude (V)	3.3 (2.5–4.3)
	- Pulse width (μs),	60 (60–90)
	- Frequency (Hz)	145 (100–185)
	Left: - Amplitude (V)	3.4 (2.2–4.3)
	- Pulse width (μs),	60 (60–90)
	- Frequency (Hz)	130 (100–185)
Location of contacts with negative polarity in relation to the midpoint of the intercommissural line	Right (mm): - Lateral	11.7 (10.4–13.1)
	- Posterior	3.4 (3.0–4.0)
	- Inferior	2.1 (1.0–5.6)
	Left (mm): - Lateral	11.4 (9.6–13.0)
	- Posterior	3.5 (3.3–5.2)
	- Inferior	2.6 (1.2–4.2)
	Intercommissural line (mm)	24.8 (23.5–25.6)

Levodopa equivalent doses calculated according to Østergaard et al. [12], and Calne [13]

### Procedure

All anti-PD medications were withdrawn the night before testing (from 10 pm) and all patients were kept as in-patients. In the morning of the study, an independent health care professional programmed the DBS to either ON or OFF. The test order of DBS ON/OFF and eyes closed/eyes open was randomized using a Latin square design, to avoid any systematic differences and bias. DBS settings were concealed to the personnel making the assessments. The posturography tests were performed at a minimum of 30 min after programming the DBS, and thus, there was at least a thirty-minute washout period between DBS ON/OFF tests. The test session was repeated in the other DBS state the same day with the assessments performed in the same eyes closed/eyes open order.

### Experimental design

**Fig. 1:**
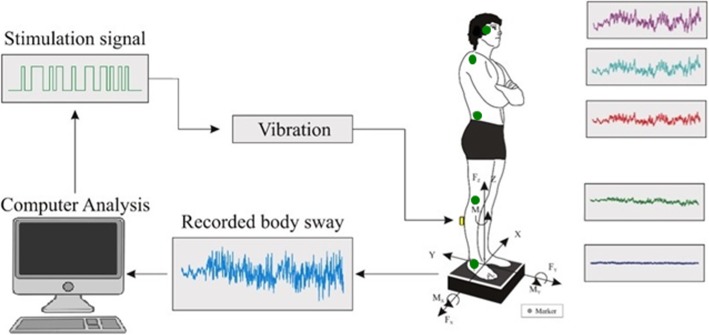
Schematic illustration of the experimental setup. While performing a standard posturography test including randomized balance perturbations, two technical methods were used for detecting tremor. A force platform was used for detecting tremor in the ground reaction forces and a 3D motion capture system was used for detecting tremor in the movements of the head, should, hip and knee. The locations of the 3D position markers are illustrated by green circles

A custom-built force platform recorded ground reaction torques and sheer forces with six degrees of freedom using force transducers with a resolution of 0.5 N. An ultrasound 3D motion capture system (Zebris™ CMS-HS Measuring System) measured movement at five anatomical proximal bony landmarks on the right side of the patient, which faced the motion detector unit, see Fig. 1. The first marker (Head) was attached to the patient’s cheekbone (os zygomaticum), the second marker (Shoulder) to the shoulder (tuberculum majus), the third (Hip) to the hip bone (crista iliaca), the fourth (Knee) to the knee (lateral epicondyle of femur), and the fifth marker (Ankle) to the anklebone (lateral distal fibula head). The markers, fastened to the selected recording sites with adhesive tape, remained attached to the patients until all assessments were completed. Each marker registered its position in three directions, i.e., anteroposterior, lateral and vertical. The measurement resolution in all directions was 0.4 mm. Blahak et al. (2007) has previously validated that the Zebris™ motion capture system can detect tremor in PD patients and that the properties of the tremor recorded correspond to those simultaneously recorded with Electromyography (EMG) [16].

The posturography test included a period of quiet stance followed by balance perturbations. Balance perturbations are commonly used in posturography tests, as the increased postural challenge enhances the ability to reveal pathologies and to display the contribution from vision for postural control [17, 18]. In this study, it was also important to ensure that throughout all assessments, the patients should always be in a mode of actively controlling their stability, and thus, the tremor recorded should be either action tremor or postural tremor. Therefore, the patients were exposed to randomized balance perturbations induced by vibrators strapped over the gastrocnemius muscles. During calf muscle vibration, the muscle spindles contained in it are activated giving rise to the proprioceptive illusion that the body is being tilted. The illusion produces postural countermeasures resulting in an opposing tilt [19]. The vibrators (6 cm long and 1 cm in diameter) had vibration amplitude of 1.0 mm and frequency of 85 Hz. Before vibration commenced, a 30-s control period of quiet stance was recorded to ensure that no frequency peaks detected were produced by the vibratory stimulation itself. The vibratory stimulations were applied as pulses, where both the vibration on and off state durations ranged from 0.8 to 6.4 s, according to a pseudorandom binary sequence (PRBS) schedule [20] during a period of 205 s making each tremor assessment 235 s long. The PRBS schedule defined the periodicity of stimulation pulses, which yielded a Fast Fourier transform (FFT) -validated effective bandwidth in the region of 0.1–2.5 Hz.

Patient testing was undertaken with eyes closed and eyes open with the order randomized. A five-minute rest period was given to the patients between eyes closed and eyes open tests. With the vibrators attached, each patient was instructed to stand in an erect and relaxed posture, barefoot on the force platform, with arms folded across the chest. The patient’s heels were 3 cm apart and feet at an angle of approximately 30° open to the front using guidelines. Patients were 1.5 m away from a wall and were instructed to focus on a 4 × 6 cm image directly ahead of them at eye level or stand with their eyes closed depending on the test condition. All patients were naive to the stimulus and were not informed about the effect calf vibration would have on their balance. The participants listened to calm classical music through closed over-ear headphones during all assessments to reduce possible movement references from external noise sources and to avoid extraneous sound distractions.

Customized computer programs controlled the vibratory stimulation and sampled the force platform data and 3D motion capture data at 50 Hz. In an offline procedure, the force platform data and 3D motion capture data were synchronized in time before the spectral analysis was performed, utilizing that both recording systems in parallel also recorded the vibratory stimulation.

Tremor in PD is usually graded using the UPDRS protocol, which is based on subjective scores between 0 and 4 categorizing the severity. For comparison, each individual patient was concomitantly scored with the UPDRS in DBS ON and DBS OFF by the same expert (specialist PD nurse or Neurologist), while this expert was blinded to the DBS state. Item 20 of UPDRS part III was used for assessing rest tremor in A) the face, lips and chin (scored 0–4), B) hands (right and left, each scored from 0 to 4), and C) feet (right and left, each scored from 0 to 4). Item 21 was used for assessing action or postural tremor in the hands (right and left, each scored from 0 to 4).

The force platform and 3D motion capture system are capable of capturing tremor of different frequencies and amplitudes and are therefore capable of capturing a range of tremor types e.g., resting, postural, action etc. and their threshold to detect and characterize the tremor will be the same. That said, the design of the posturography setup meant that the technical recordings could not be used for unequivocally distinguishing whether the tremor was resting, postural and action tremor during the posturography tests.

### Data analysis

**Fig. 2:**
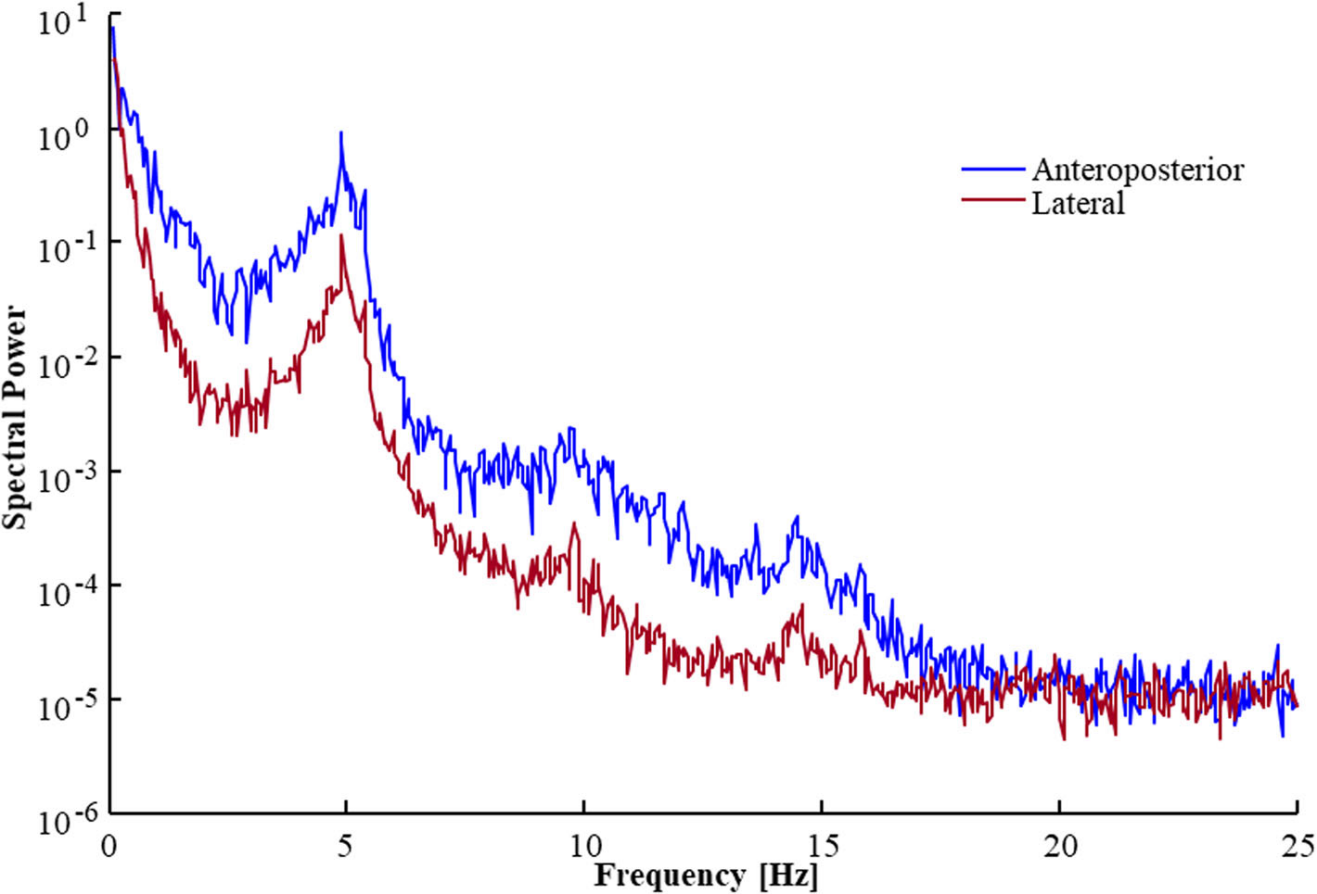
Anteroposterior and lateral spectral power patterns in recorded torque (reflecting COP position) by a force platform, from a patient standing with eyes open with DBS OFF. The patterns were almost identical, including that both were influenced by a tremor peak of an identical frequency. However, the anteroposterior spectral power levels about 10 times larger than the lateral power levels in the frequency range below 20 Hz. Note that after the normalization the values on the y-axis will have no unit

This study focused on tremor peaks detected in the anteroposterior direction. However, as illustrated in Fig. 2, with few exceptions tremor peaks were simultaneously detected in the lateral and anterior directions at an almost identical peak frequency.

The spectral FFT power analyses were performed by a custom-made program Postcon™ using the spectral analysis module implemented in Labview™ 2018 [21]. Before the FFT analysis, the data from the force platform and 3D Motion systems were normalized to account for anthropometrical differences between patients. Hence, the force platform FFT analysis was performed on raw recorded torque data (reflecting center of pressure (COP) position) normalized with the patient’s weight and height, with the unit and scale including normalization of [(N m Kg^− 1^ m^− 1^)·100]. The 3D Motion FFT analysis was performed on raw data normalized with the patient’s height, with the unit and scale including normalization of [m 10^− 3^ m^− 1^]. The measurement data was converted into FFT samples reflecting the spectral power in the frequency range from 0.1–25 Hz.

Problems associated with interpreting a spectral analysis of tremor when multiple peaks are present are known [22]. In our spectral analysis of tremor peaks, we wanted to exclude any non-PD related harmonics or side bands. To do this, we excluded peaks below 4.2 Hz and above 7.5 Hz as per convention [22, 23]. The tremor peaks were quantified in a semi-manual process by an expert investigator (author PF). The inclusion criteria were that a tremor peak should have 1) a distinctively higher spectral power peak (at least 200% larger) than the adjacent spectral power activity and 2) the spectral power increase should have clearly defined upper and lower spectral boundary limits. Tremor peaks fulfilling the inclusion criteria were manually marked by the operator with a cursor in a log-scaled plotting window. The software thereafter presented the exact spectral power amplitude and frequency for the marked tremor peak. In cases where multiple frequency peaks could be detected, commonly due to harmonic resonances to the primary tremor peak, the largest (i.e., primary) tremor peak was selected.

Additionally, the average spectral power was calculated for three frequency bands: 0.1–4 Hz; 4–7 Hz and 7–25 Hz to illustrate how DBS ON/OFF and vision affected the recorded spectral power during the tests. Performing a wide spectrum power analysis together with a tremor analysis has been recommended to isolate effects due to intervention or experimental state (DBS or eyes open/eyes closed) [22].

### Statistical analysis

The pair-wise comparisons determining the respective roles of DBS and visual states on spectral power were performed with Wilcoxon matched-pairs signed-rank test (Exact sig. 2-tailed) [24]. Because of the case study design and the asymmetrical distribution of the tremor peaks found across test conditions, no statistical analyses were performed on peak tremor characteristic. Hence, the tables and figures serve primarily to illustrate the kind information and its detail level, which can be obtained from the two objective technical methods used for assessing the properties of the tremor in this study. Non-parametric statistics were used in the statistical evaluation as not all data sets were normally distributed before or after logarithmic transformation. In the pair-wise Wilcoxon comparisons a *p*-value < 0.017 were considered statistically significant after performing appropriate Bonferroni corrections. The statistical analyses were performed using IBM SPSS 24.0 software.

## Results

### Manual assessment of tremor using the UPDRS protocol

When using the clinically standard UPDRS method, visible Parkinsonian tremor in DBS OFF state was detected in six of ten patients, though only distally in the hands and feet, see Table 2. Tremor was not detected with the UPDRS in four of ten patients. With DBS ON, the evaluator was not able to detect tremor using the UPDRS.

**Table 2:** UPDRS values

UPDRS part III scores, without anti-PD medication	DBS turned OFF	DBS turned ON
20: Face lips	0.2 (0.6)	0.0 (0.0)
20: Right hand	0.9 (1.2)	0.0 (0.0)
20: Left hand	0.8 (1.1)	0.0 (0.0)
20: Right foot	0.6 (1.0)	0.0 (0.0)
20: Left foot	0.7 (1.2)	0.0 (0.0)
21 Action right hand	0.2 (0.4)	0.0 (0.0)
21 Action left hand	0.6 (1.0)	0.0 (0.0)
Item 20 & 21 (tremor)	3.9 (4.5)	0.0 (0.0)
Total UPDRS Score	48.0 (15.7)	20.6 (6.1)

(SD) values are presented

UPDRS part III: Unified Parkinson’s disease Rating Scale, motor examination. The maximum total score on the UPDRS part III is 108 points, and higher scores reflect more severe motor symptoms.

Item 20 of UPDRS part III assesses rest tremor in A) the face, lips and chin (scored 0–4), B) hands (right and left, each scored from 0 to 4), and C) feet (right and left, each scored from 0 to 4). Item 21 assesses action or postural tremor in the hands (right and left, each scored from 0 to 4).

Without medication: Overnight withdrawal of all anti-Parkinsonian medication for 10–12 h. All individuals were on L-dopa, and seven out of the ten participants were also on dopamine agonists (ranging from 20 to 50% of L-dopa equivalent dose). When tested, all participants experienced clinical off symptoms.

The UPDRS assessments were done at the same occasion as the physical assessments of tremor.

### Technical assessment of tremor

Three patients, including the two with the most severe tremor were not able to complete the entire posturography assessments with DBS OFF while standing with eyes closed because of balance instability and that they required external support in standing. Data from these three patients prior to test termination were included in the analysis.

The two technical methods were able to detect tremor in the same 6 subjects as per the UPDRS and in 3 patients where tremor was not identified with the UPDRS. Tremor peaks were detected in force platform recordings in 6 of 10 patients and with the 3D motion capture system in 7 of 10 patients in DBS OFF. The tremor detection of the two measurement systems did not perfectly overlap, and thus, tremor was detected by either of the measurements systems in 9 of 10 subjects. Hence, although tremor was common in our PD group, it was not present in all patients despite an average disease duration of 18 years. With DBS ON, we were still able to detect tremor in force platform recordings and 3D motion capture system in 1 of 10 patients and with the 3D motion capture system in 3 of 10 patients. Thus, we detected tremor with DBS ON using either technical method but not the UPDRS in 3 of 10 subjects.

**Fig. 3:**
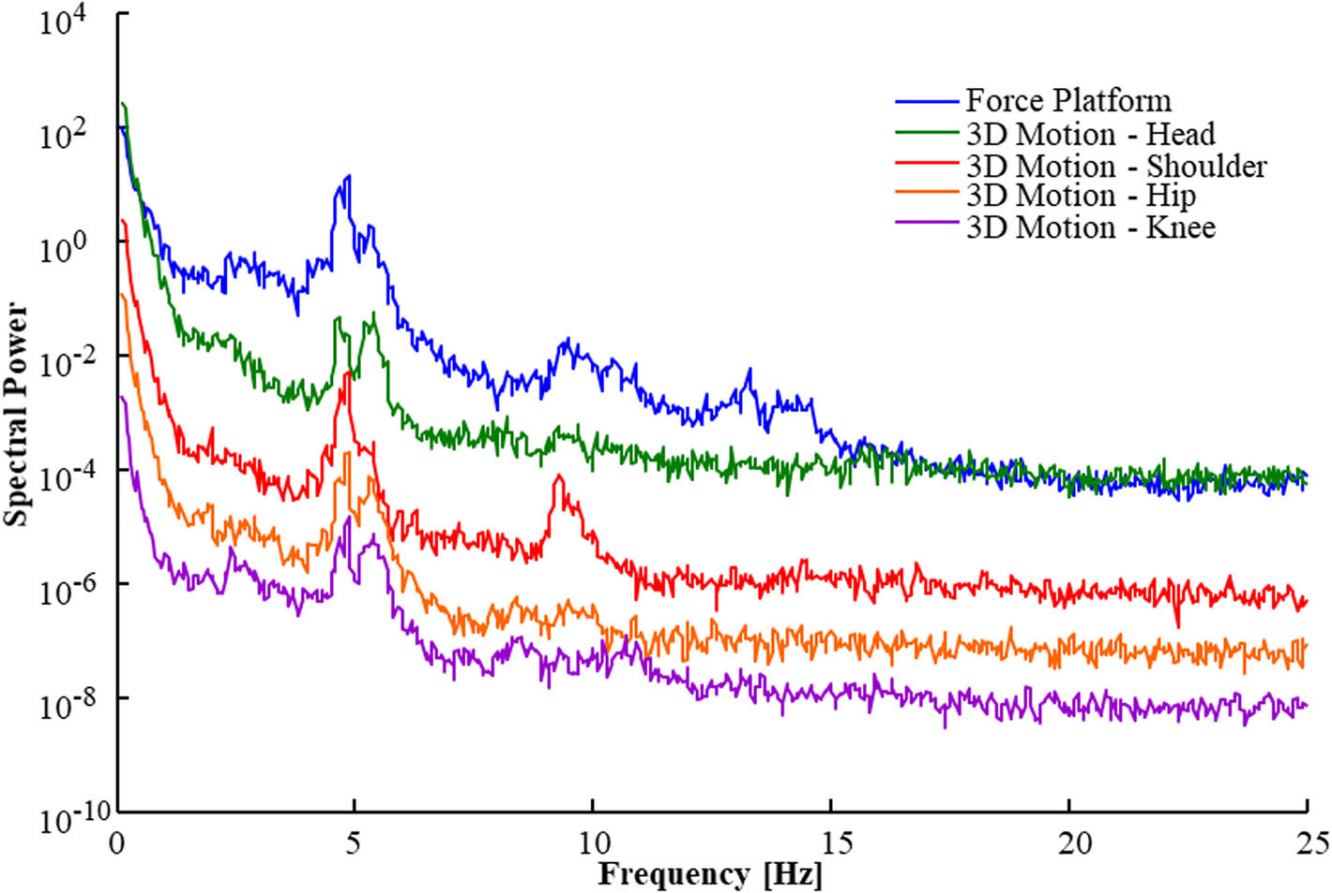
Parkinsonian tremor peaks detected in anteroposterior direction in a patient standing with eyes closed and DBS OFF. Noteworthy, both the force platform and 3D motion capture system detected tremor peaks, and the 3D motion capture system detected tremor peaks at all four multi-segmental body positions of an identical frequency. Both assessment methods record physical representations of the tremor, though of different entity, i.e., as additional torque towards the support surface or as additional body movements within a specific frequency range. The power spectrums have been moved vertically in the figures to better display the individual recordings

The tremor, if any was detected, had a strong peak typically within the 4.5–5.5 Hz range, but the peak frequency could range between 4.3 to 7.3 Hz, and the tremor influenced a spectral bandwidth of 1 Hz or more around the peak frequency. Furthermore, in cases of high amplitude Parkinsonian tremor, peaks were captured with both recording methods and at all body sites. The high amplitude tremor had almost identical peak frequency across all body sites, albeit with different amplitudes, see Fig. 3.

Fifty tremor peaks were detected by the force platform and 3D motion capture system during the four test conditions. The force platform was slightly better at detecting Parkinsonian tremor peaks (33% detections in all tests made) than the 3D motion capture system (23%), recording tremor at 4 body sites, see Table 3. The 3D motion capture system tended to detect slightly more tremor peaks at the shoulder than at the head, knee and hip. In most cases (69%), more than one body site was affected by tremor, see Table 4.

**Table 3:** Tremor peaks detected with the two assessment methods

PD tremor detected with recording method [%]		Posturography test			
		DBS OFF - EC	DBS OFF - EO	DBS ON – EC	DBS ON - EO
**Force platform**		50	60	10	10
**Body movements**	**Head**	40	30	0	20
	**Shoulder**	30	50	10	20
	**Hip**	30	30	10	10
	**Knee**	20	30	20	20

**Table 4:** Tremor propagation

Tremor propagation	Number of segments with tremor if tremor was found			
Tremor found [%]	**1**	**2**	**3**	**4**
	31	25	25	19

Eighty-five percent of the tremor peaks detected in force platform recordings and 70% of tremor peaks detected in 3D motion analysis were identified during DBS OFF, see Table 5. This means that only 15% of the tremor peaks detected in force platform recordings and the 30% detected in 3D motion analysis were identified during DBS ON. Activating the DBS removed 86.5% of the tremor peaks identified during DBS OFF with the force platform, whereas changing from standing with eyes closed to eyes open removed 8.5% of the tremor peaks identified with the force platform. Activating the DBS removed 66% of the tremor peaks perceived during DBS OFF with the 3D motion system, whereas changing from eyes closed to eyes open removed 14.5% of the tremor peaks identified with the 3D motion system. Both recording systems detected somewhat more (16%) tremor peaks with DBS OFF with eyes open compared to eyes closed.

**Table 5:** Tremor peaks detectable after changing DBS state or vision

Test properties			PD tremor		
**Recording method**	**DBS State**	**Vision**	**Detected tremor Peaks**	**Remained with changed vision [%]**	**Remained with changed DBS [%]**
**Force platform**^**a**^	OFF	Closed	5	100	20
		Open	6	83	17
	ON	Closed	1	100	100
		Open	1	100	100
**Body Movements**^**b**^	OFF	Closed	12	92	25
		Open	14	79	43
	ON	Closed	4	100	75
		Open	7	57	86

^a^The maximum number of tremor peaks possible to detect was *n* = 10

^b^The maximum number of tremor peaks possible to detect was *n* = 10 patients 4 body sites =40

### DBS and tremor change

**Fig. 4:**
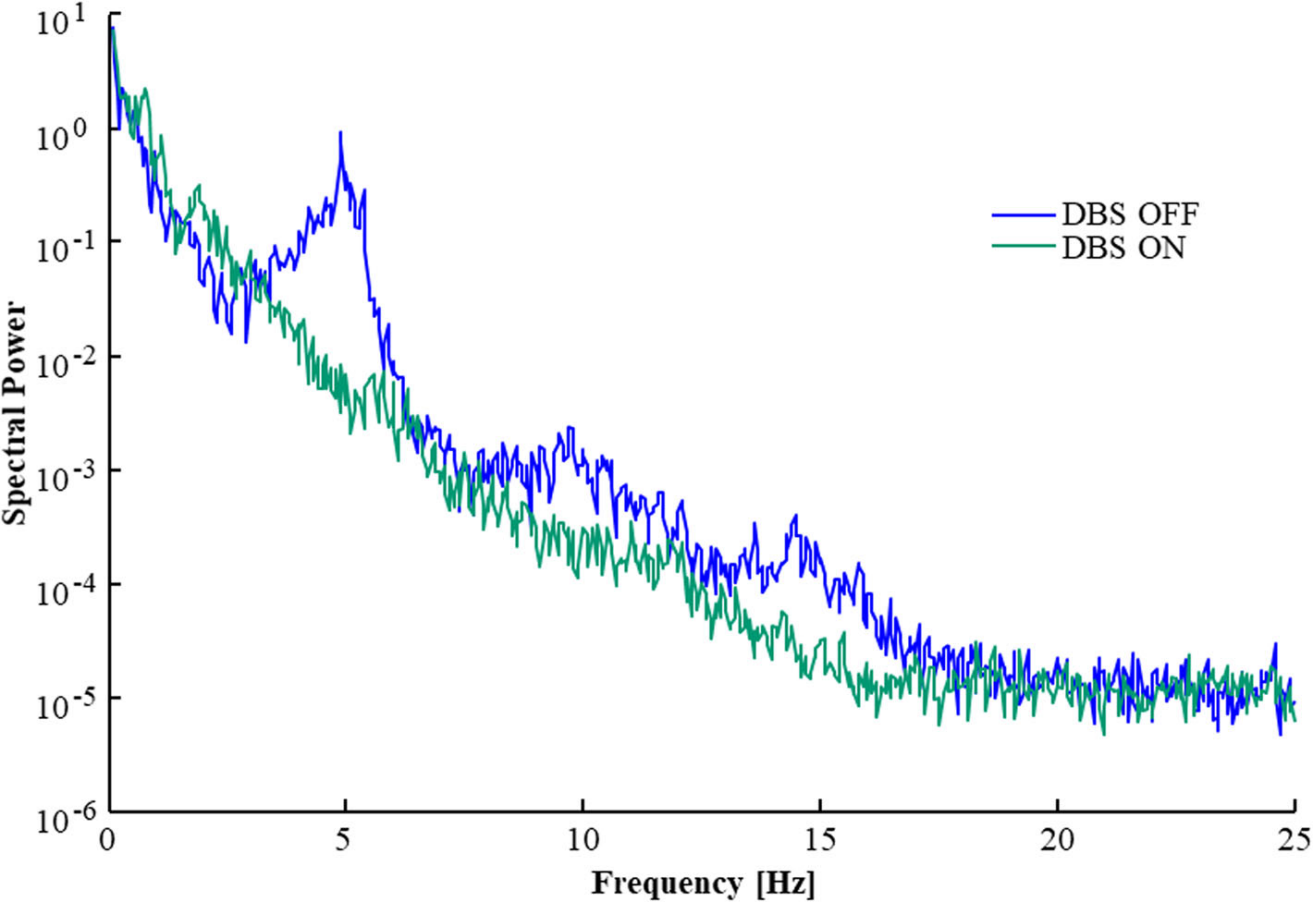
Spectral power of Parkinsonian tremor with DBS OFF and ON, when recorded by a force platform, from one patient standing with eyes open. Note how turning the DBS ON generally lowers also the spectral power within the 4–10 Hz range and slightly increase the spectral power in the 0.1–4 Hz range. Moreover, the DBS suppress both the primary Parkinsonian tremor at 5 Hz and the harmonics at 10 Hz and 15 Hz frequencies

With eyes closed, DBS ON reduced the average spectral power of Parkinsonian tremor peaks by 96% when recorded with a force platform, and by 93% when recording body movements, see Table 6 and Fig. 4. Similarly, with eyes open, DBS reduced the average spectral power of Parkinsonian tremor peaks by 97% when recorded with a force platform, and by 97% when recording body movements. The peak frequency of the Parkinsonian tremors was not notably affected by DBS activation in any condition.

**Table 6:** Tremor frequency and spectral power changes when altering DBS state

Changes from DBS OFF to DBS ON				
**Recording method and site**	**Vision**		Frequency increase [Hz]*	**Peak power reduction**^**a**^
**Force platform**	Closed		0.0 (0.0)	24.5 (19.9)
	Open		0.0 (0.0)	35.1 (22.1)
**Body Movements**	Closed	Head	0.0 (0.0)	2.0 (1.4)
		Shoulder	0.0 (0.0)	36.6 (1.0)
		Hip	0.0 (−)	3.3 (−)
		Knee	0.0 (0.0)	15.2 (14.6)
	Open	Head	−0.6 (0.5)	12.3 (5.7)
		Shoulder	−0.3 (0.3)	45.8 (22.8)
		Hip	0.0 (0.0)	66.4 (46.6)
		Knee	0.1 (0.1)	19.6 (9.4)
**Body Movements - Average all sites**	Closed		0.0 (0.0)	14.3 (8.0)
	Open		−0.2 (0.2)	36.1 (21.1)

^a^Mean (SEM) values are presented for tremor frequency increase (frequency _OFF_ - frequency _ON)_ and peak power increase (power _OFF_ / power _ON_) for the different vision states. In the cases no tremor peak was detected in one state, the frequency change was set to 0 and the power changes was calculated using the power at the frequency where the peak tremor was detected at in the other vision state

### Vision and tremor change

With DBS OFF, visual input (eyes open) reduced the average spectral power of Parkinsonian tremor peaks by 29% when recorded with a force platform and by 1% when recorded with a motion capture system, see Table 7. With DBS ON, visual input reduced the average spectral power of Parkinsonian tremor peaks by 58% when recorded with a force platform and by 52% when recorded with a motion capture system.

**Table 7:** Tremor frequency and spectral power changes when altering visual input

Changes from Eyes Closed to Eyes Open				
**Recording method and site**	**DBS**		**Frequency increase [Hz]**^**a**^	**Peak power decrease**^**a**^
**Force platform**	OFF		0.0 (0.1)	1.4 (0.6)
	ON		0.1 (−)	2.4 (−)
**Body Movements**	OFF	Head	0.1 (0.1)	1.8 (1.4)
		Shoulder	0.1 (0.1)	0.7 (0.2)
		Hip	0.3 (−)	0.7 (−)
		Knee	0.0 (0.0)	0.8 (0.6)
	ON	Head	−0.7 (0.7)	0.9 (0.1)
		Shoulder	0.1 (−)	2.4 (−)
		Hip	0.1 (−)	3.0 (−)
		Knee	0.1 (0.4)	2.0 (1.5)
**Body Movements - Average all sites**	DBS OFF		0.1 (0.1)	1.0 (0.8)
	DBS ON		−0.1 (0.5)	2.1 (0.8)

^a^Mean (SEM) values are presented for tremor frequency increase (frequency _EC_ -frequency _EO)_ and peak power increase (power _EC_/power _EO_) for the different DBS states. In the cases no tremor peak was detected in one state, the frequency change was set to 0 and the power changes were calculated using the power at the frequency where the peak tremor was detected at in the other DBS state

### Manifestations of tremor at different body sites

**Fig 5:**
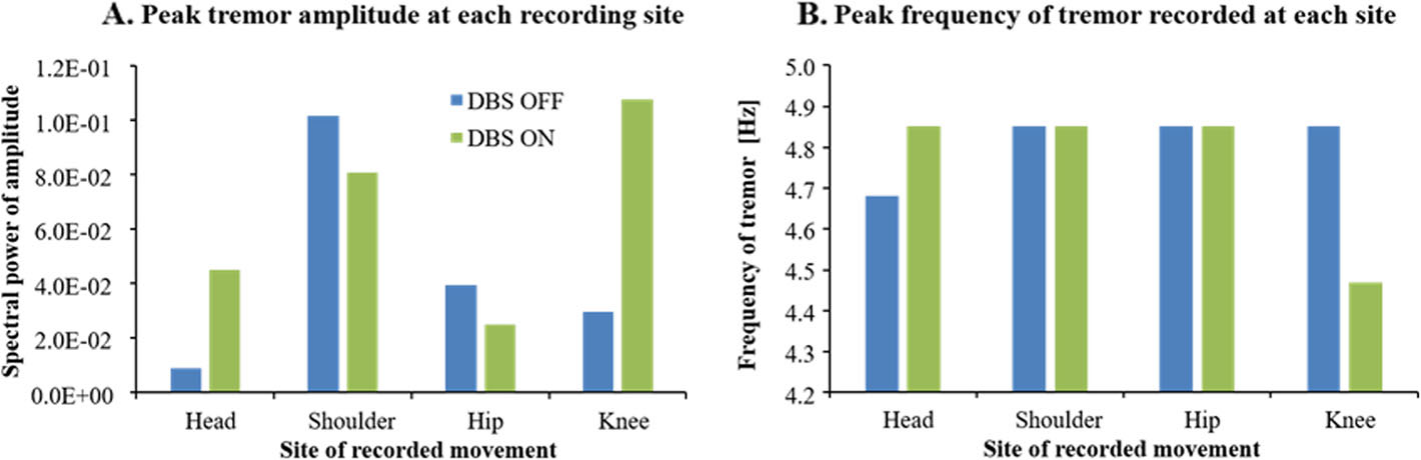
**a** Tremor amplitudes during DBS OFF and DBS ON, in terms of spectral power, at each recording site in one patient with tremor simultaneously in all body sites assessed. For graphical reasons the amplitude of tremor during DBS ON has been multiplied with 100. The data recorded are from when the same patient is standing with eyes open. **b**, Tremor peak frequency recorded at each site in the same patient and test condition as in (**a**)

The typical body movement pattern during posturography with calf vibration is that of an inverted pendulum, i.e., the movements increase fairly linearly from the feet up. The peak amplitudes of the Parkinsonian tremor increased with increasing sizes of body movements up to the shoulder level, after which the tremor amplitude made a marked drop in amplitude when recorded at the head. This is illustrated in Fig. 5a using data from a representative patient. Figure 5a also illustrates that DBS in this patient reduced shoulder and hip tremor more than the head and knee tremor. The tremor peak frequency was altered by less than 0.4 Hz between recorded body sites, as shown in Fig. 5b.

### DBS and spectral power reduction

**Fig. 6:**
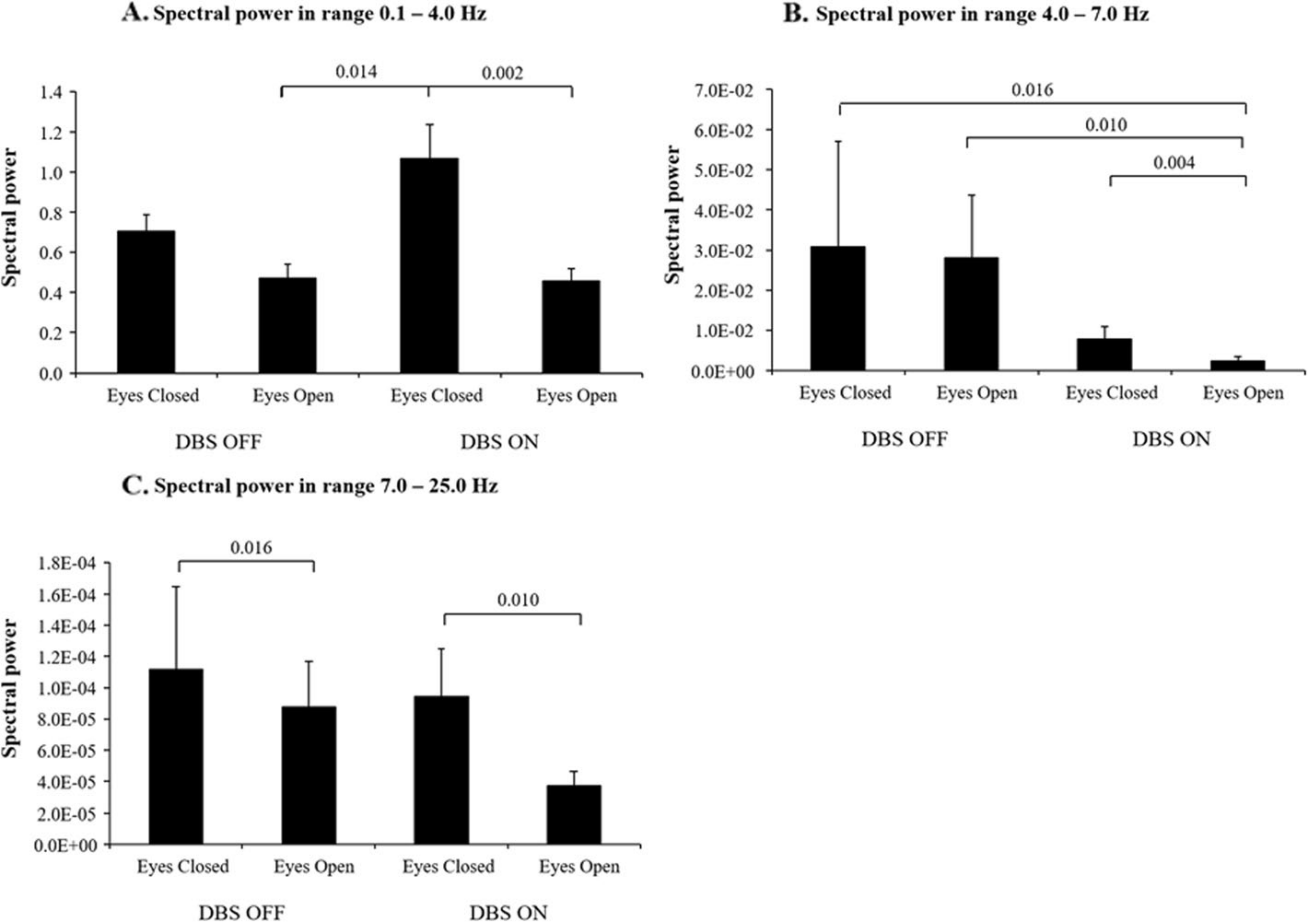
Mean spectral power and SEM values in force platform recordings for spectral ranges **a**: 0.1–4.0 Hz; **b**: 4.0–7 Hz; and **c**: 7–25 Hz during the different DBS states and vision states. The in several cases extremely high SEM values is figures B and C are produced by patients with exceptionally strong tremor peaks

Visual input (eyes open) improved stability more with DBS ON than with DBS OFF, see Fig. 6. With DBS OFF, visual input reduced the spectral power by 33% on average in the 0–4 Hz spectrum (NS), by 53% in the 4–7 Hz spectrum (NS), and by 53% in the 7–25 Hz spectrum (*p* = 0.002). With DBS ON, visual input reduced the spectral power by 57% on average in the 0–4 Hz spectrum (*p* = 0.002), by 69% in the 4–7 Hz spectrum (*p* = 0.004) and by 61% in the 7–25 Hz spectrum (*p* = 0.010).

### Technical tremor detection boundaries with a force platform and 3D motion analysis system

The force platform had a spectral white noise limit of about 10·10^− 6^ [(N m Kg^− 1^ m^− 1^)·100] usually appearing above 20 Hz. The amplitude of the smallest Parkinsonian tremor detected was 12.6·10^− 3^ [(N m Kg^− 1^ m^− 1^)·100], see Table 8 for all tremor test condition details. The 3D-Motion capture system had a white noise limit of about 30·10^− 6^ [m 10^− 3^ m^− 1^] usually appearing above 10 Hz. The amplitude of the smallest Parkinsonian tremor detected was 161·10^− 6^ [m 10^− 3^ m^− 1^]. For both recording systems, the smallest tremor amplitudes accepted to be included in the analysis were at least four times larger than the recording device’s spectral white noise levels.

**Table 8:** Tremor frequencies and peak spectral power amplitudes for PD tremor

Tremor characteristics				PD tremor	
**Recording method**	**DBS State**	**Vision**	**Body Site**	**Peak Frequency (Hz)**	**Power**^**a**^**(10**^**−3**^**)**
**Force platform (Nm)**	OFF	Closed^b^	–	4.6 (0.2)	467.4 (434.8)
		Open	–	4.7 (0.1)	408.3 (245.2)
	ON	Closed	–	5.0 (−)	152.4 (−)
		Open	–	4.9 (−)	63.8 (−)
				**Peak Frequency (Hz)**	**Power**^**a**^**(10**^**−6**^**)**
**Body Movements (mm)**	OFF	Closed^b^	Head	4.7 (0.2)	396.2 (72.8)
			Shoulder	4.9 (0.1)	5858.5 (2372.8)
			Hip	5.0 (−)	278.1 (−)
			Knee	4.9 (−)	7916.5 (−)
		Open	Head	4.6 (0.1)	4676.6 (2351.2)
			Shoulder	4.7 (0.1)	22,916.7 (19,768.4)
			Hip	4.7 (0.1)	15,192.9 (12,276.1)
			Knee	5.7 (0.8)	11,758.8 (8975.5)
	ON	Closed	Head	–	–
			Shoulder	5.0 (−)	1922.6 (−)
			Hip	5.0 (−)	745.0 (−)
			Knee	5.1 (0.2)	2025.9 (1750.8)
		Open	Head	5.6 (0.7)	612.1 (163.0)
			Shoulder	5.6 (0.7)	605.1 (204.1)
			Hip	4.9 (−)	248.9 (−)
			Knee	5.0 (0.6)	797.4 (278.2)
**Body Movements - Average all sites**	OFF	Closed	–	4.9 (0.1)	3612.3 (122.8)
		Open	–	4.9 (0.1)	13,636.7 (16,622.8)
	ON	Closed	–	5.0 (0.1)	1173.3 (875.4)
		Open	–	5.3 (0.7)	565.9 (215.1)

^a^The force platform FFT analysis was performed on raw data (recorded torque reflecting COP position) normalized with the patient’s weight and height with the unit and scale after normalization of [(N m Kg^−1^ m^−1^) 100]. The 3D motion capture FFT analysis was performed on raw data (position) normalized with the patient’s height with the unit and scale after normalization of [m 10^−3^ m^− 1^]

^b^The three patients with the poorest stability, including two patients that were among those with the strongest recorded tremors under the other test conditions, could not perform this test entirely

## Discussion

The aim of this methodologies case study was to investigate the utility of a 3D motion capture system and a posturography force platform to detect tremor in patients with PD. Hence, we wanted to determine whether these recording methods were sensitive enough to detect whether STN stimulation (without anti-PD medication) and visual input (eyes open / eyes closed) affect the amplitude and peak frequency of tremor at different body sites. We also wanted to detail how tremor in PD manifests across body segments with STN stimulation ON and turned OFF. Using a 3D motion capture system, proximal tremor was detected across all body segments with non-uniform amplitude characteristics across sites of the body but with an almost identical peak frequency. Moreover, DBS-STN reduced Parkinsonian tremor at all body segments, though the tremor reduction was not uniform across body sites.

Tremor is an approximately sinusoidal oscillation of any part of the body and both the amplitude and frequency can vary. Moreover, the primary Parkinsonian tremor, e.g., at 5 Hz, often causes harmonics at 10 Hz, 15 Hz and 20 Hz frequencies (i.e., at 2, 3 and 4 multiples of the main tremor frequency). Our findings show that the amplitude and frequency of tremor are not always proportional to the anatomic position of the body, e.g., according to single link pendulum models of the human body, particularly with DBS ON (Table 8) [25]. Moreover, oscillating neural activity may become clinically relevant despite the motor output amplitudes being below visual threshold levels. The somatosensory systems might still be activated by the neural activity and thus produce sensations of motion or distortions manifested as shaking or vibration in the body [26]. Prior reports suggest that the internal tremor sensations [26] could be caused by a physical movement from subclinical muscle activity producing a tremor that cannot be observed visually [27, 28] but can be detected with advanced technical recording methods [6–8].

### Motion analysis of Parkinsonian tremor

In this study, we captured Parkinsonian tremor in 7 of 10 PD patients with the 3D motion capture system and in 6 of 10 PD patients with the force platform. We found that the typical Parkinsonian tremor generally had a strong peak in the 4.5–5.5 Hz range, and the tremor influenced a spectral bandwidth of 1 Hz or more around the peak frequency. Furthermore, in cases of intense Parkinsonian tremor were near identical in frequency at all sites, see Fig. 3a & Fig. 5a. However, in the case illustrated in Fig. 5a, tremor spectral amplitude increased with increasing sizes of body movements up to the shoulder level, after which the tremor amplitude was markedly lower in amplitude when recorded at the head. This finding suggests that Parkinsonian tremor amplitudes are related to or modulated by topographical levels, i.e., by the anatomical position of the body. Hence, conducting the extended UPDRS part III assessment across different levels of the body could be considered a standard clinical approach, such as determining the tremor at the head, the upper extremities and the lower extremities.

### Vision and tremor reduction

Most tremors were detected by our technical methods when vision had reduced the baseline postural control activity, i.e., most Parkinsonian tremors were detected during DBS OFF with eyes open. This finding suggests that certain tremors are not added on top of other concurrent physical movement activities but have fixed amplitude, and thus, can be hidden by other concurrent spectral activity. Moreover, we also found that the tremor peak amplitudes decreased on average by about 55% more with eyes open compared with eyes closed. This observation is in line with reports where relatively small increases in feedback loop gains (including central reflex loops) are capable of inducing a large amplitude tremor [29, 30]. Thus, the increased neurological motor drive gains required to maintain stability with eyes closed might influence the amplitude of the tremor peaks but not the tremor frequency. Noteworthy three patients, including the two with the largest tremor amplitudes, could not complete the posturography assessments during calf vibration with DBS OFF while standing with eyes closed. However, all patients were able to complete the posturography tests with eyes open, suggesting that the destabilizing effects from Parkinson’s disease on postural control to some extent can be addressed with visual feedback. This finding shows that the contributions of DBS and vision might interact. Such interaction effects on tremor could however not be explored in this study as DBS ON almost always removed all signs of tremor.

### Technical challenges of quantitative assessment of tremor

Based on our results, several technical recommendations can be made for tremor analysis in clinical settings:

#### Equipment

First and foremost, recording devices must be sufficiently sensitive to pick up small amplitude tremor. In our study, the smallest tremor peaks detected had normalized spectral amplitudes of 12.6·10^− 3^ [(N m Kg^− 1^ m^− 1^)·100] for the force platform and 161·10^− 6^ [m 10^− 3^ m^− 1^] for the 3D motion capture system. For both recording systems, the smallest tremor amplitudes accepted in the analysis were at least four times larger than the recording device’s spectral white noise levels. As there is a large cost, in terms of the physical energy needed to move large masses fast, the physical presentation of neurological tremor might be of small movement amplitudes. This effect is because high-frequency forces will be suppressed by the double integrator dynamics present in the Newtonian dynamics of the causal dependence of force variables resulting in the position variables. Hence, tremor of small amplitude may still represent a central neurological disorder of impact.

High-quality force platforms can detect Parkinsonian tremor well, and this device has also been shown able to detect strong forms of orthostatic tremor active in the 12–18 Hz frequency range [31]. Another method to detect tremor is by recording EMG activity in muscles and looking for rhythmic activity [16, 32, 33]. Blahak et al. has validated that Parkinsonian tremor simultaneously recorded by both EMG and 3D motion capture system was similar in terms of its characteristics [16]. However, a potential limitation with the EMG recording technique is that it may be difficult to translate recorded EMG activities into physical movement amplitudes.

#### Assessment procedures

More tremor peaks were detected when vision reduced baseline postural control activity, i.e., most Parkinsonian tremors were detected during DBS OFF with eyes open. This finding suggests that certain tremors can be masked by other movement activities. Moreover, longer continuous recording periods producing more data allows the FFT algorithms to better refine and detail the spectral content and reduce the influence of random recording noise. In this study, the continuous recordings were 230 s long for each test condition. Furthermore, performing the assessments during posturography with balance perturbations may facilitate detecting tremor as increases in motor feedback loop gains can induce larger tremor amplitudes [30].

#### Signal analysis

The established analysis method to detect tremor is time series analysis using FFT [33]. However, in order to obtain values that describe a tremor in a way that can be compared between individuals or patient categories, it is also vital to normalize the measured data for anthropometrical differences between patients. Thus, the data from the force platform were normalized with the patients’ mass and height and data from the 3D motion capture system were normalized with the patients’ height. Secondly, the FFT algorithms should be meticulously designed to suppress measurement artefacts so these are not allowed to obscure the final FFT diagram, e.g., by suppressing random recording noise. Finally, a semi-manual analysis step might be necessary to remove spectral peaks from harmonic manifestations and to determine that a tremor peak has: 1) distinctively higher spectral power peak (at least 200% larger) than the adjacent spectral power activity and 2) the spectral power increase should have clearly defined upper and lower spectral boundary limits.

#### Result presentation

Due to the large change in spectral power within the frequency range of interest when analyzing body movements in upright standing, most Parkinsonian tremors can only be detected in an FFT diagram by using a logarithmic scale on the power axis.

### Limitations

One limitation of this study was that the recordings were performed on a relatively small but well-defined group where not all subjects had a strong and potentially clinically relevant tremor. Thus, studies on larger patient groups are warranted including patients with varied forms of tremor and tremor locations. Moreover, new studies should also aim to define relationships between recorded physical characteristics of a tremor (e.g., tremor amplitude, location etc.) and the tremor’s clinical relevance. That said, such studies need to be preceded by studies such as this to determine the technical equipment and methodology for tremor detection and research.

Another limitation of this study was that the 3D motion capture system did not score the tremor at the same physical position as was done with UPDRS or with the force platform. Thus, discrepancies can be expected between the data from the 3D motion capture system and UPDRS, in that the more proximal placement of the 3D motion capture markers meant that they had to detect tremor of smaller amplitude than was required by the UPDRS. The two technical methods were able to detect tremor in all 6 of the ten subjects mimicking the UPDRS, but also in 3 additional subjects.

One limitation could potentially be that the wash-out period between DBS ON and OFF states was in the range of 30 min or longer, thus in some cases residual DBS effects might remain. To minimize any systematic effects, the order of DBS ON and OFF was therefore randomized across the test group. Moreover, when checking our data for potential after-effects from a previous DBS ON we could find none, though some effects on the amplitude cannot be excluded, e.g., if the PD subject first performed tests with DBS ON and displayed a tremor, a tremor was always also detected in the following test performed with DBS OFF.

Finally, one could argue that technology measures usually requires additional time for data clearance and analyses, which can act as a barrier for its implementation in clinical practice. The way forward could be using wearable technology in the home setting to monitor PD tremor continuously, which could also apply for postural control measures.

## Conclusions

This study revealed that both a high accuracy force platform and a 3D motion analysis system could detect the effects of DBS and visual input, but also that the 3D motion system was more versatile in that it could detail the presence and properties of tremor at individual body sections. The latter proved relevant as the manifestation of tremor varied between segments, i.e., the tremor amplitude detected increased with increasing sizes of body movements up to the shoulder level, after which the tremor amplitude was markedly lower in amplitude when recorded at the head. The tremor detection of the two measurement systems did not perfectly overlap, and thus, tremor was detected by either of the measurements systems in 9 of 10 subjects. Technical recording methods provide objective and sensitive measures of tremor such as peak amplitude, frequency and distribution pattern that can guide treatments. A major benefit with the technical systems used in this study is that they provide repeatability, precision and reliability, independent of operator or clinic where it is used. Subjective scoring may vary in its accuracy and definition of scoring thresholds, e.g., how large should the tremor be to reach level 2 in UPDRS may differ between trained experts. Furthermore, as detailed in the values presented in Tables 3, 4, 5 and 7, technical systems can detail the tremor characteristics to a much higher physical resolution and range, i.e., the 4-level UPDRS assessment can be replaced with technical scales with resolutions of 500 scale levels or more. Hence, as illustrated in Table 6, a technical system shows that turning ON the DBS while the patient has eyes open produces a 35.1% amplitude reduction of the tremor detected in the ground reaction forces. Therefore, technical methods may offer a more sensitive option than the UPDRS to detect disease progression and small amplitude tremor with the typical characteristics for PD.

## Data Availability

All data generated or analyzed during this study are included in this published article.

## Abbreviations

3D: 3 dimensional
CNS: Central nervous system
COP: Center of pressure
DBS: Deep brain stimulation
EMG: Electromyography
FFT: Fast Fourier transform
GPI: Globus Pallidus Internus
PD: Parkinson’s disease
PRBS: pseudorandom binary sequence
STN: Subthalamic nucleus
UPDRS: Unified Parkinson’s Disease Rating Scale
